# Standardized Health data and Research Exchange (SHaRE): promoting a learning health system

**DOI:** 10.1093/jamiaopen/ooab120

**Published:** 2022-01-17

**Authors:** Sierra Davis, Louis Ehwerhemuepha, William Feaster, Jeffrey Hackman, Hiroki Morizono, Saravanan Kanakasabai, Abu Saleh Mohammad Mosa, Jerry Parker, Gary Iwamoto, Nisha Patel, Gary Gasparino, Natalie Kane, Mark A Hoffman

**Affiliations:** 1 Children’s Mercy Research Institute, Children’s Mercy Hospital, Kansas City, Missouri, USA; 2 Department of Pediatrics, Children’s Hospital Orange County, Orange, California, USA; 3 Department of Emergency Medicine, Truman Medical Centers, Kansas City, Missouri, USA; 4 Department of Biomedical and Health Informatics, University of Missouri Kansas City, Kansas City, Missouri, USA; 5 Department of Pediatrics, Children’s National Hospital, Washington, District of Columbia, USA; 6 Clinical Research Systems, Indiana University Health System, Indianapolis, Indiana, USA; 7 Research Informatics, University of Missouri, Columbia, Missouri, USA; 8 Department of Internal Medicine, University of New Mexico, Albuquerque, New Mexico, USA; 9 Cerner Enviza, Cerner Corporation, Kansas City, Missouri, USA

**Keywords:** electronic health record, data sharing, data science, learning health system

## Abstract

Aggregate de-identified data from electronic health records (EHRs) provide a valuable resource for research. The Standardized Health data and Research Exchange (SHaRE) is a diverse group of US healthcare organizations contributing to the Cerner Health Facts (HF) and Cerner Real-World Data (CRWD) initiatives. The 51 facilities at the 7 founding organizations have provided data about more than 4.8 million patients with 63 million encounters to HF and 7.4 million patients and 119 million encounters to CRWD. SHaRE organizations unmask their organization IDs and provide 3-digit zip code (zip3) data to support epidemiology and disparity research. SHaRE enables communication between members, facilitating data validation and collaboration as we demonstrate by comparing imputed EHR module usage to actual usage. Unlike other data sharing initiatives, no additional technology installation is required. SHaRE establishes a foundation for members to engage in discussions that bridge data science research and patient care, promoting the learning health system.

## BACKGROUND AND NEED

Data captured by electronic health record (EHR) systems during the delivery of healthcare are widely recognized as a valuable source of clinical phenotypic information,^1^ outcomes data, and as a resource for health services research.^2^ Recent innovative research based on EHR data has demonstrated the potential for deep learning and other data science methods to glean new insights from “real-world” clinical data.^3^ A key limitation of much EHR work, however, is that it is limited to the EHR of a single organization.

Although significant research has been performed using EHR data from individual organizations,^4^ this work is often limited by a lack of geographic, demographic, or health practice diversity. Several initiatives have sought to connect disparate EHRs to address the limitations of working with data from a single institution. For example, the electronic Medical Records and Genomics (eMERGE) network has federated data from member sites and has successfully contributed to new insights in cardiovascular disease and other conditions.^5^ However, the work to harmonize a local phenotype to the eMERGE phenotype algorithms can require significant effort per category and may not be feasible for many organizations.^6^

**Figure 1.**
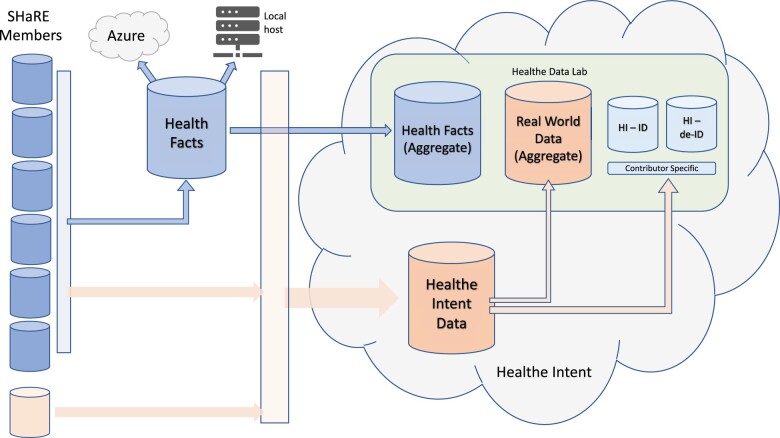
Data flow between SHaRE member source systems, Health Facts and Healthe Intent. HI-ID: Healthe Intent identified; HI-de-ID: Healthe Intent de-identified.

An alternative approach to federated networks is to aggregate EHR data into a warehouse or data lake. While many organizations using EHR systems have implemented local data warehouses, there are few cross-institutional data warehouses primarily comprised of EHR data. Cerner Corporation has operated a large de-identified aggregate data warehouse, Health Facts^®^ (HF) since 2000 (Figure 1). Cerner stopped adding new data to HF 2018 and has recently updated their data sharing approach to a new architecture, Cerner Real-World Data (CRWD).^7^ CRWD operates similarly to HF but uses a contemporary cloud-based architecture, incorporates additional data elements, and includes a rapidly growing number of organizations. HF and CRWD are voluntary initiatives in which Cerner clients agree to the inclusion of de-identified, Health Insurance Portability and Accountability Act (HIPAA)-compliant data extracted from their Cerner EHR in aggregate data warehouses. CRWD contributors are not limited to organizations using a Cerner EHR if those organizations are using other Cerner capabilities, for example, population health. The data include patient demographics, diagnoses, billing, medication, and laboratory data as well as many discrete patient observations including vital signs. HF data have been used to support research related to infectious disease management, cancer, cardiology, and neurology.^8–12^ While de-identified data cannot be validated, the frequency of diagnosis codes in HF is generally consistent with data in the National Inpatient Survey (NIS).^13^

By design, the identities of the sites participating in HF and CRWD are masked in distributions of the data, limiting opportunities to collaborate and perform comparative quality improvement initiatives. We report an initiative among HF and CRWD organizations to establish a framework that resolves these limitations and promotes a learning health system—the Standardized Health data and Research Exchange (SHaRE).

## SHaRE MEMBER RECRUITMENT AND GOVERNANCE

Candidate organizations were identified through two means. First, Children’s Mercy collaborated with Cerner Corporation (Kansas City, MO) to invite organizations known by Cerner to have received a copy of the HF data and that contribute data into the HF resource or the CRWD initiative. Additionally, Children’s Mercy contacted organizations known to have recently published findings based on their use of HF data^14^ or participation in CRWD. Eligible organizations contribute to HF or CRWD, have an active data use agreement (DUA) with Cerner, and did not have competitive concerns with other candidate members. The participating organizations have already implemented the data extraction processes required to load data into HF or CRWD. Likewise, Cerner has implemented the de-identification and data mapping processes for participating organizations. Candidates also already have access to the full data of HF and/or CRWD; no new data transfers were necessary.

Candidate organizations were invited to join SHaRE with the intended benefits of enhancing the value of their existing access to HF and CRWD, providing access to other unmasked member organization identifiers (IDs), delivering the first 3 digits of zip code (zip3) information for patients treated at SHaRE organizations and offering a platform for collaborative knowledge sharing. Of the 10 organizations initially invited to participate, 7 completed the participation agreement and became founding participants. The other 3 organizations expressed an intent to participate in the future. The results of this session informed a participation agreement. The 7 organizations that completed the participation agreement prior to the submission of this manuscript are deemed the founding participants of SHaRE: Children’s Hospital Orange County (CHOC), Children’s Mercy Hospital (CMH), Children’s National Hospital (CNH), Indiana University Health System (IUHS), Truman Medical Center (TMC), University of Missouri (MU), and University of New Mexico (UNM).

In their agreement, participating organizations provided information about their data implementation (cloud or local hosted) and use a variety of local or cloud-hosted options. The participating organizations access the HF data through 3 general strategies. Some have installed the data in a locally managed resource. Others have implemented a cloud-hosted approach using either Microsoft Azure (Microsoft, Seattle, WA) or Amazon Web Services (AWS). Some organizations access the data through HealtheDataLab (HDL), a Cerner-managed data science platform which can be provisioned with CRWD and/or HF data. CRWD is only available through HDL and is not distributed for independent installation.

The SHaRE participation agreement is approved by Cerner legal and executed between CMH and each participating organization. The agreement commits organizations to handling site unmasking information and zip3 data under the requirements of their DUA with Cerner. Publications intended to identify participating organizations by name require the express approval of the SHaRE representative associated with that organization. Publications describing occurrence of health conditions and patterns of healthcare delivery at the zip3 level are allowed with no restrictions. Publishing comparative outcomes research requires approval of any organizations with patient zip3 data included.

After completing the agreement, participating organizations provided the SHaRE operations group at Children’s Mercy with their organization and facility IDs from HF and/or their tenant IDs from CRWD, and technical details about how they access the data.

## CHARACTERIZATION OF SHaRE MEMBER DATA CONTRIBUTION

We characterized the volume and geographic diversity of data contributed to HF and CRWD by founding SHaRE organizations. The Children’s Mercy team performed queries on the HF December 2017 version using RStudio Server Pro 1.3.1056-1 with R version 3.6.1 hosted on Azure (Microsoft, Redmond, WA). The HF July 2018 version and Q3 September 2020 release of CRWD were queried using PySpark version 2.4.4 hosted in HDL.^7^

SHaRE organizations consist of stand-alone pediatric hospitals and multifacility academic medical centers (Table 1). Collectively, the cumulative available HF data contributed by these organizations represent 4.8 million unique patients and 63 million encounters; the cumulative available CRWD data contributed by SHaRE members through early 2021 represent 7.4 million patients and 119 million encounters. Current SHaRE members care for approximately 7% of the patients in HF and 8% in CRWD.

**Table 1.** Health facts and CRWD data contributed by SHaRE participants

Category	HF facilities	HF patients	HF encounters	CRWD patients	CRWD encounters
Stand-alone pediatric hospitals	28	1 889 488	19 407 635	2 900 947	22 548 160
Multifacility academic medical center	23	2 882 351	44 012 503	5 594 173	87 324 258

*Note:* Health Facts (HF) includes data from all SHaRE members except Children’s Hospital Orange County. Cerner Real-World Data (CRWD) patients and encounters represent all 7 founding SHaRE members.

**Figure 2.**
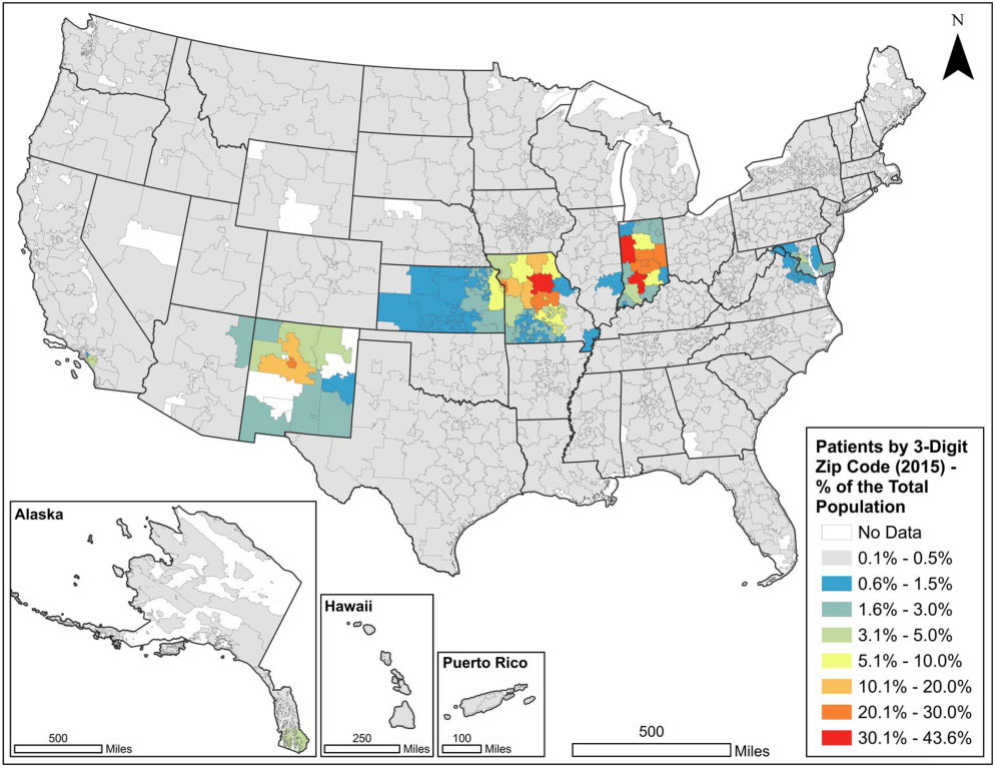
Patients treated at 7 SHaRE organizations in Cerner Real-World Data in 2015 as a percent of the total population in the 2010 census.

Cerner generated an enhancement file for SHaRE that contains the zip3 of the de-identified patients associated with participating organizations. Cerner excludes patients residing in zip3 regions that do not meet the de-identification standards (eg, a minimum population of 20 000 people) of the HIPAA. We used 2015 patient data from CRWD and the 2010 Decennial Census to map an annual estimate of patients per the total population by zip3. We retrieved 2010 total population counts for the 5-digit zip code tabulation area (ZCTA) geography from the US Census Bureau API using the tidycensus R package.^15^ The population counts from SHaRE organizations were grouped by the first 3 digits of the ZCTA and summed to estimate the total population by zip3. The number of SHaRE CRWD patients in 2015 by zip3 was then divided by the zip3 total population estimates to control for population density. This created an indicator of the distribution of SHaRE patients by zip3 as a percent of the total population. The final indicator was joined with a zip3 layer produced by Esri^16^ and mapped using Esri ArcGIS Pro v. 2.5.0. (Redlands, CA) (Figure 2).

**Figure 3.**
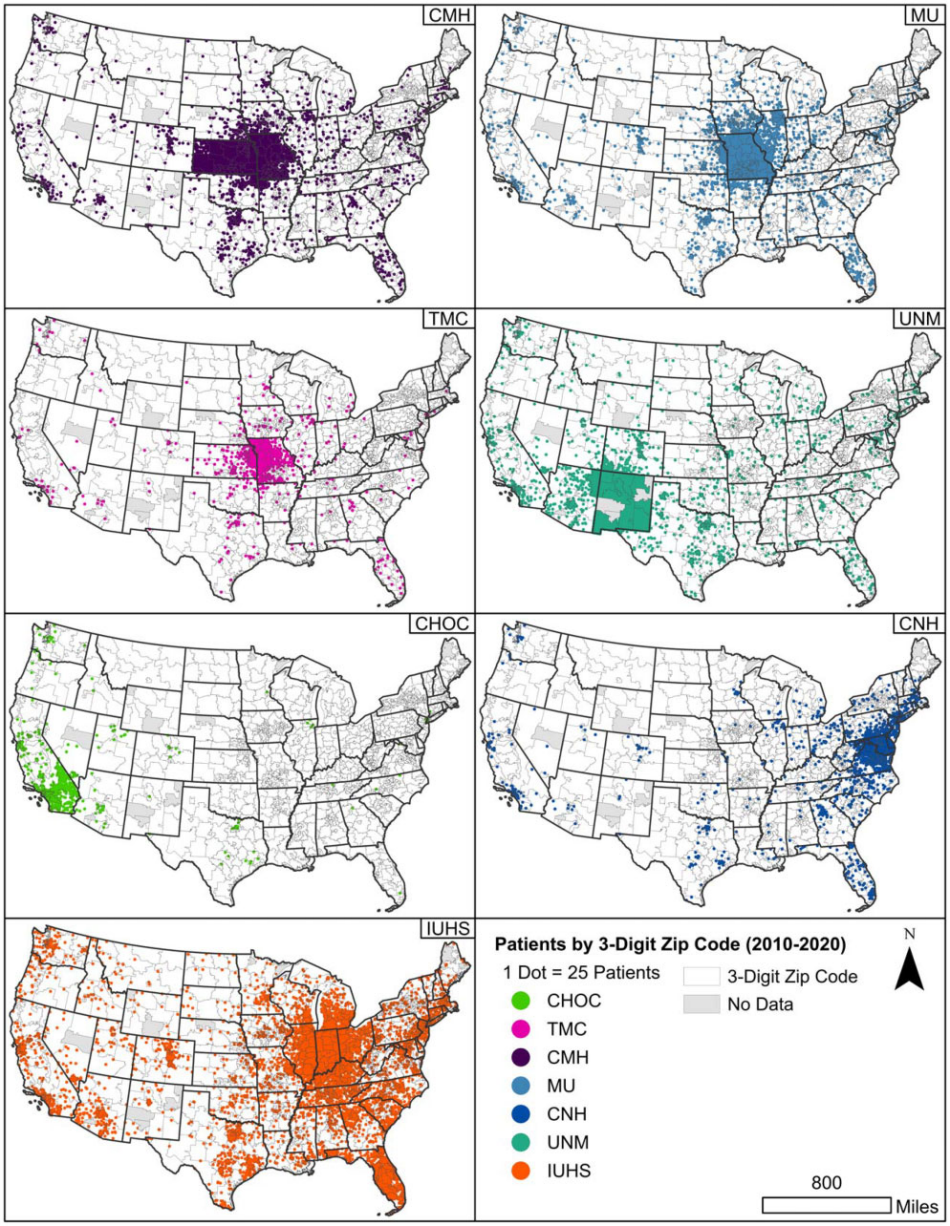
Dot density of unique patients by zip3 for 7 SHaRE organizations in Cerner Real-World Data (2010–2020). One dot represents 25 patients in a zip3 region. Abbreviations: CHOC: Children’s Hospital Orange County; CMH: Children’s Mercy Hospital; CNH: Children’s National Hospital; IU: Indiana University Health System; MU: University of Missouri; UNM: University of New Mexico; TMC: Truman Medical Center.

We mapped the number of unique patients by zip3 using the Dot Density tool in ArcGIS Pro for samples of CRWD to visualize patient data by location. Figure 3 presents the dot density of unique patients by SHaRE organization in CRWD for 2010–2020 with a panel for each of the 7 SHaRE organizations. Three organizations, CMH, TMC, and MU, have overlapping service areas. The count of unique patients by zip3 is represented by dots randomly distributed within each zip code, where 1 dot represents 25 patients. For HF, zip3 information was only available for 4 SHaRE organizations in the July 2018 HF data. Of the 3 298 724 patients contributed to HF by these organizations, 17.4% had zip3 data between 2002 and 2018.

## DEMONSTRATION OF BENEFIT

In order to demonstrate the value of the organization-level unmasking, we validated inferences performed in a paper that imputed the use of EHR modules (Microbiology, Pharmacy, Surgery) without access to the contributing organizations.^17^ We asked SHaRE participants to confirm the use of EHR modules imputed in the analysis of HF contributor heterogeneity. If the use or nonuse of a Cerner module matched between the imputed status (in use, not in use) and the response of the SHaRE organizations contributing to HF, the entry is scored as correct; otherwise, it is scored as incorrect (Table 2). The results indicate that the EHR module utilization logic has a classification accuracy score of 90% and is statistically significant (*P* value .009).^18^ SHaRE members can now incorporate this into future work to exclude the 3 incorrect site level imputations.

**Table 2.** Imputed and actual EHR module installation

Organization	Micro	Micro sus	Pharmacy	Surg case	Surg procedure
Children’s Mercy	•	•	•	•	□
Children’s National	•	•	•	▪	▪
Indiana University Medical System	•	•	•	•	o
Truman Medical Center	•	•	•	•	o
University of Missouri	•	•	•	•	o
University of New Mexico	o	o	•	•	o

*Note:* •: in use, correct; o: not in use, correct; ▪: in use, incorrect; □: not in use, incorrect.

## DISCUSSION

Aggregate EHR data provide a useful resource for multisite quality improvement and research. Many data sharing initiatives require substantial effort from each participating site to harmonize terminologies to a Common Data Model (CDM), making participation challenging. The SHaRE consists of a group of organizations that rely on a common EHR vendor to aggregate, de-identify, and standardize data from a national cohort of HF and CRWD contributors. HF and CRWD contributors reflect the diversity of healthcare in the United States, including larger, often academic, organizations with the capacity to harmonize to a CDM and organizations that lack these resources. The latter often serve underrepresented populations and are a key differentiator for these data resources.

SHaRE participants already had access to data extracted from EHR systems through participation in an established data collaboration with their EHR vendor, Cerner. The complex work of de-identifying the data while maintaining longitudinal relationships between patient encounters is performed by Cerner using patient IDs that remain consistent across encounters. Likewise, Cerner standardizes incoming data to terminologies including ICD 9, ICD 10, SNOMED, LOINC, NDC, and CPT if the data were not already harmonized through local mapping at the contributor. Each organization had already completed the challenging legal and organizational work to participate in HF and/or CRWD. Each organization has access to a full copy of HF, CRWD, or both and are able to independently query the full data set. Cerner makes CRWD available in the Observational Medical Outcomes Partnership (OMOP) format.^19^

The masking of contributing organization identities is an appropriate policy of the data aggregator, Cerner, but restricts dialogue between contributors. SHaRE resolves these limitations by enabling participating members to safely unmask their organization IDs to one another through a secure portal managed by CMH. The portal provides a file showing the mapping between HF organization ID or CRWD tenant ID and SHaRE member organization names. The patient data remain fully de-identified. The SHaRE participant agreement establishes appropriate use of the information released to SHaRE organizations and the requirement to handle the unmasking matrix as sensitive data.

We demonstrated the benefits of facility-level unmasking by reviewing the accuracy of recently imputed EHR module (lab, pharmacy, surgery) usage. Other features in HF and CRWD reflect local implementation decisions that can be clarified through the communication channels opened by SHaRE. SHaRE will enable more significant clinical conversations about workflows and processes that promote improved outcomes and a learning health system and will provide opportunities to understand and mitigate source system heterogeneity in aggregate EHR data.^17^^,^^20^

Our evaluation of the scope and spatial extent of HF and CRWD data contribution by SHaRE contributors shows that SHaRE organizations have served patients from most zip3 regions in the United States. The density of patients in the regions served by SHaRE matches expected patterns. IUHS and the MU serve a particularly high percentage of patients in their regions. CHOC, in the greater Los Angeles area, serves a smaller percent of the population but has patients from a wide area in Southern California.

With SHaRE, it is now possible for participating organizations to perform an analysis and then open a dialogue with other members to compare specific practices. Furthermore, the inclusion of zip3 data is a significant improvement compared to the facility-level census region included in standard HF distributions. With zip3 data, it is possible to evaluate the epidemiology of conditions, their correlation with social determinants, and to conduct health services research.

Vendor provided EHR data aggregation, whether Cerner CRWD or Epic COSMOS,^21^ have an important role to play in the broader landscape of the Learning Health System. While limited to their respective customer ecosystems, these resources offer a path to including organizations without the capacity to independently perform data harmonization. SHaRE demonstrates the capacity of a subgroup of data contributors to add value to these systems by establishing distinct benefits, such as greater geo-precision and contributor-level unmasking while preserving patient privacy. SHaRE members are engaged in collaborative efforts to harmonize Cerner-provided data mappings related to patient demographics and other topics.

## FUNDING

The organizational meeting for SHaRE was funded by Centers for Disease Control and prevention Cooperative Agreement NU47OE000105-01-01. HM is supported by the National Institute of Health (NIH) NCATS Clinical and Translational Awards Program, UL1TR001876. The contents are solely the responsibility of the authors and do not necessarily represent the view of CDC or NIH.

## AUTHOR CONTRIBUTIONS

MAH conceptualized SHaRE and drafted the manuscript. SD performed HF queries in support of the manuscript, contributed to the content, and coordinated with member sites. JP and NP contributed to the content and design of the participation agreement and to the review of the manuscript. NK generated the maps and contributed to the review of the manuscript. LE, WF, JH, HM, SK, ASMM, GI, and GG contributed to the review and editing of the manuscript and to the organizational design of SHaRE.

## ETHICS APPROVAL

Ethics approval and consent to participate—Work with Health Facts and CRWD is considered nonhuman subjects.
